# Lactate-mediated neural plasticity genes emerged during the evolution of memory systems

**DOI:** 10.1038/s41598-022-23784-8

**Published:** 2022-11-10

**Authors:** Amal Bajaffer, Katsuhiko Mineta, Pierre Magistretti, Takashi Gojobori

**Affiliations:** 1 Computational Bioscience Research Center (CBRC), King Abdullah University of Science and Technology (KAUST), Thuwal, 23955-6900 Saudi Arabia; 2 Biological and Environmental Science and Engineering Division (BESE), King Abdullah University of Science and Technology (KAUST), Thuwal, 23955-6900 Saudi Arabia; 3 Computer, Electrical and Mathematical Sciences and Engineering Division (CEMSE), King Abdullah University of Science and Technology (KAUST), Thuwal, 23955-6900 Saudi Arabia; 4 Research Organization for Nano and Life Innovation, Waseda University, Tokyo, 162-0041 Japan

**Keywords:** Computational biology and bioinformatics, Evolution, Neuroscience

## Abstract

The ability to record experiences and learning is present to different degrees in several species; however, the complexity and diversity of memory processes are cognitive function features that differentiate humans from other species. Lactate has recently been discovered to act as a signaling molecule for neuronal plasticity linked to long-term memory. Because lactate is not only an energy substrate for neurons but also a signaling molecule for plasticity (Magistretti and Allaman in Nat Rev Neurosci 19:235–249, 2018. https://doi.org/10.1038/nrn.2018.19), it is of particular interest to understand how and when memory-related genes and lactate-mediated neural plasticity (LMNP) genes emerged and evolved in humans. To understand the evolutionary origin and processes of memory and LMNP genes, we first collected information on genes related to memory and LMNP from the literature and then conducted a comparative analysis of these genes. We found that the memory and LMNP genes have different origins, suggesting that these genes may have become established gradually in evolutionarily and functional terms and not at the same time. We also found that memory and LMNP systems have a similar evolutionary history, having been formed with the gradual participation of newly emerging genes throughout their evolution. We propose that the function of LMNP as a signaling process may be evolutionarily associated with memory systems through an unidentified system that is linked by 13 common genes between memory and LMNP gene sets. This study provides evolutionary insight into the possible relationship between memory and the LMNP systems that deepens our understanding of the evolution of memory systems.

## Introduction

Memory is the process that allows humans to retain information over time^2^. The physiological properties of memory have been widely examined, revealing that the function of memory is conserved across species of vertebrates and invertebrates^3^. Nevertheless, it is challenging to interpret the evolution of memory, focusing only on the physiological data. Besides, a number of studies that have concentrated on the evolution of a memory system is limited^3^. Functionally, memory systems require the involvement of many genes^4^; comprehensive research of memory-related genes is required due to the accumulation of genomics and relevant data to understand the origin and evolution of memory^3^.

Pellerin and Magistretti^5^ proposed that lactate released by astrocyte can be a functional energy source for neurons, through a mechanism known as the astrocyte-neuron lactate shuttle (ANLS); later Magistretti and colleagues showed that lactate is an intercellular signaling molecule by effectively stimulating the expression of some neural plasticity genes that are involved in the process of memory formation^6,7^. Lactate is a metabolic intermediate that various species can aerobically and anaerobically produce in their tissues, and is a known energy source for humans, providing energy not only to muscles, but also to the heart and brain^8^. Although the human brain constitutes only 2% of total body weight, the metabolic demands of the brain consume about 20% of the total oxygen consumption in the human body^9^, and the energy required for appropriate brain function represents approximately 25% of the body’s entire glucose usage^10^. As a signaling molecule, lactate changes the redox state of neurons once it is converted to pyruvate and also increases the intracellular NADH/NAD ratio. Consequently, it potentiates the activity of the N-methyl-D-aspartate receptor (NMDAR) to raise the calcium influx inducing the expression of neural plasticity-related genes^7,11^. Besides, lactate has signaling features through the hydroxycarboxylic acid receptor 1 (HCAR1). Scavuzzo et al.^12^ showed the involvement of HCAR1 signaling in memory consolidation.

Previous studies have revealed the evolutionary conservation of ANLS in mice^5,6^ and invertebrates. Lactate is produced from astrocytes by metabolizing glucose from cerebral blood vessels directly or from glycogen storage through glycolysis or glycogenolysis, respectively. Then, it is exported to neurons and can be used as a source of energy for neural activities through oxidative phosphorylation in the mitochondria^5,13,14^. In Drosophila, for example, glial cells produce lactate and alanine from pyruvate to fuel mitochondria in neurons, indicating that metabolic coupling between glial cells and neurons is a global mechanism^15^. In the adult brain of Drosophila, an increasing energy influx in the neural mushroom body has been described, which is the primary center of learning and memory in insects sufficient for LTM formation^16^. Nonetheless, there is still a lack of fundamental understanding of the fluxes of energy-mediated molecules in the brain of Drosophila^17^.

The evolutionary origin of lactate dehydrogenase (LDH), an enzyme that produces lactate, has been intensively studied. Cristescu et al.^18^ suggested that L-Ldh evolved through multiple events of gene duplication, and its evolutionary history shared among vertebrates and invertebrates. Skory^19^ examined 26 LDH subunits, focusing on two subunits LDHA and LDHB from numerous hosts, including mammals, birds, fish, fungi, bacteria, and plants. The evolution of LDHA and LDHB have been previously studied, suggesting that LDHA and LDHB evolved from a single ancestral gene^20,21^. It has been showed that the production of lactate by LDH is not limited in non-human animals, but it also observed in almost all organisms, from bacteria to humans^19^. Duncan et al.^22^ discovered that some bacteria of the human colon produce lactate in low concentrations and can convert this lactate into butyrate, which is crucial for colonic health.

To date, the function of lactate as a signaling molecule promoting neural plasticity and memory has only been detected in mice^7^. It remains to be established when these memory and LMNP systems evolved. In this study, we aim to explore and understand the evolutionary origin and processes of memory-related genes, including LMNP genes. To this end, we collected orthologs in different taxonomic groups using memory and LMNP-related genes. Then, we conducted a comparative analysis of gene gain and loss of the orthologs of memory and LMNP genes to elucidate how LMNP-related genes were incorporated into the memory system. Lastly, we studied the evolutionary alteration of the functional groups of the orthologs of those genes to understand the evolutionary processes of the memory and LMNP-related genes.

## Results and discussion

### Memory and LMNP-related genes

We conducted a literature survey by reviewing a total of 572 papers, 136 of which were not accessible and therefore not considered. We selected the papers that contained a list of more than ten genes, which resulted in 15 reviews (Supplementary Table S1). However, we excluded one review (Molecular basis of pharmacotherapies for cognition in Down Syndrome) because it was unclear if the genes were related to memory. Thus, we obtained 335 memory-related genes from the 14 published reviews. We excluded six of the memory-related genes (Dcx, Plec, Cit, Gdi1, ryr3, and Dlgap1) from the analysis because they had more than one euNOG orthologous cluster, as well as 27 memory-related genes because they did not have orthologs in humans. Therefore, the total number of memory-related genes used in this study is 302 genes.

With regards to LMNP genes, we obtained 395 genes after omitting either one of 18 overlapping gene pairs between 1 and 6 h-data sets, 12 ncRNA (non-coding RNA), and one rRNA (ribosomal RNA). Seven LMNP genes (Kdm6b, Fras1, Agbl3, Met, Apaf1, Usp43, and Stk36) had more than one euNOG orthologous cluster, which we excluded from the analysis. We also removed from the analysis two LMNP proteins (Cygb and Hdac9) that were not detected by the eggnog mapper tool. Therefore, the total number of LMNP genes used in this study is 386 genes.

For the reference, we collected 23,004 sequences from the mouse proteome. The eggnog mapper detected 22,574 reference sequences, 430 proteins were not found in the mapper, and no orthologous cluster or result was found. We excluded from the analysis 322 proteins that had more than one euNOG, 26 proteins that did not have an euNOG orthologous cluster, and 1505 proteins that did not have orthologs in humans. Therefore, we used a total number of 20,721 mouse amino acid sequences as references in this study.

### Overlapping genes between memory and LMNP genes

We collected 302 and 386 genes for memory and LMNP in mice, respectively. We compared these genes to detect any commonality between the memory and LMNP systems, giving 13 genes that are common between memory and LMNP systems, namely Arc, Junb, Bdnf, Kcna6, Egr1, Nr4a1, Atf4, Fos, Itga3, Bcl2l11, Hcn1, Adcyap1, and Cckbr. Some of the 13 genes have been previously detected to be required for memory formation in a lactate-dependent manner. Lactate induces the expression of several neural plasticity genes including (Arc, c-Fos, BDNF, and Egr1) that are involved in the process of LTM formation^7^. The orthologous distribution of these genes is presented in Supplementary Table S2. The existence of overlapping genes may reflect the presence of an unidentified system that links the memory and LMNP systems. Interestingly, all these proteins have orthologs in humans and mice, suggesting that they must be essential in both systems. Further studies are needed to investigate what the unknown system might be that connects these two systems.

### Species distribution of memory and LMNP genes

We successfully detected memory-associated and LMNP genes based on our extensive literature review. We examined species from the following different taxonomic groups: vertebrates (*Homo sapiens, Mus musclus, Gallus gallus and Danio rerio),* invertebrates *(Ciona intestinalis, Drosophila melanogaster, Nematostella vectensis, Trichoplax adhaerens,* and *Amphimedon queenslandica),* Choanoflagellate (*Monosiga brevicollis*), and fungi *(Saccharomyces cerevisiae).* We based this on the variation in their taxonomic group to ensure an extensive coverage of the tree of life and because their complete proteomics are publically available.

**Figure 1:**
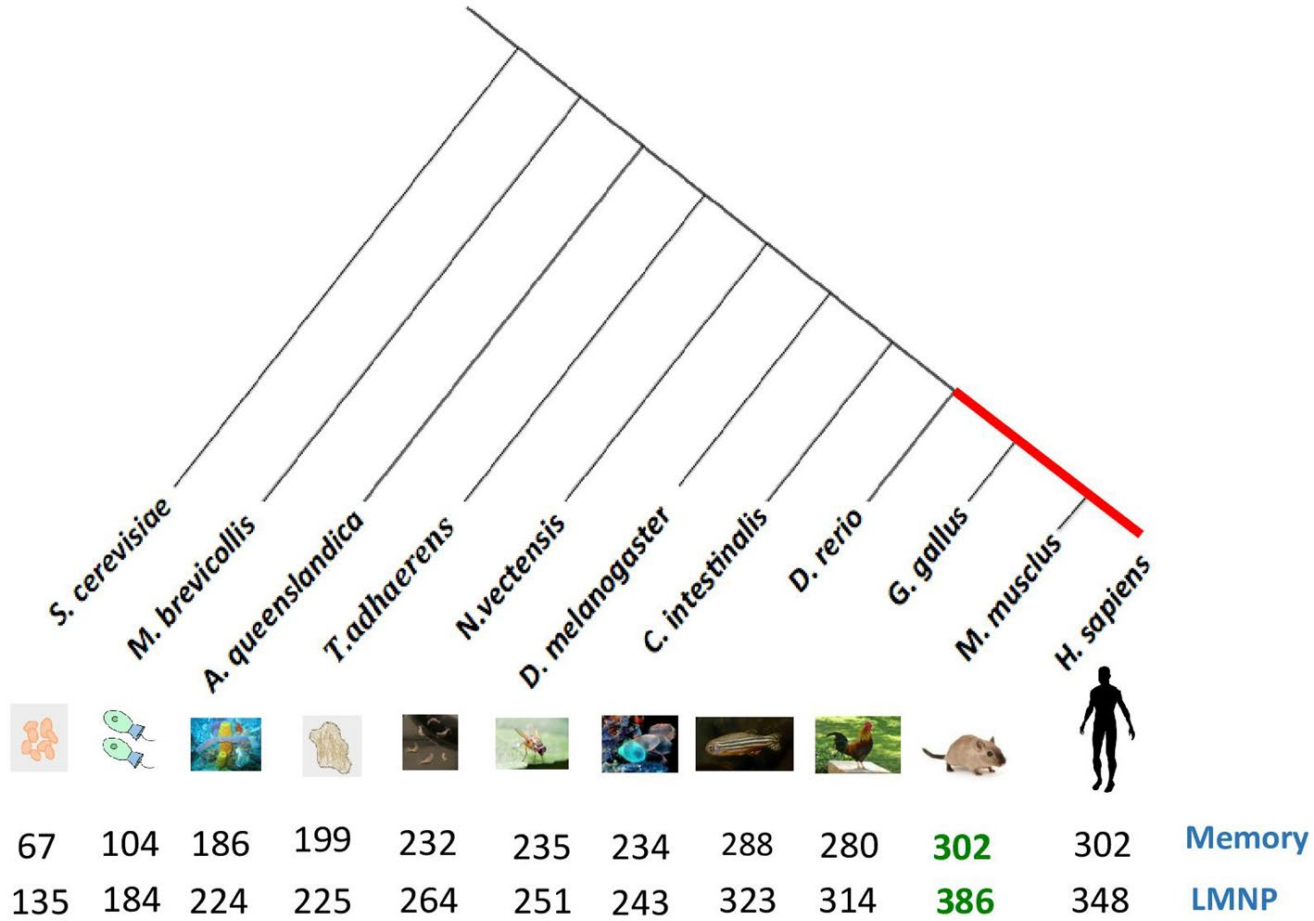
Orthologous detection of mouse memory-related genes and LMNP-related genes. The green color indicates the number of memory and LMNP-related genes in *M. musculus*, and the black color shows the orthologs of those genes. The red line represents species of vertebrates.

As seen in Fig. 1, vertebrates possess a substantial number of orthologous genes compared to other species in both systems. We detected 302 memory-related genes in mice, with the same number of orthologs of memory-related genes in humans. We detected 280 genes in chicken and 288 in zebrafish. The number of memory-related genes decreases significantly in invertebrates, choanoflagellate, and yeast compared to vertebrates (Fig. 1). This is consistent with our understanding that memory in vertebrates is more developed than in other species. One of the possible explanations could be that the memory system in vertebrates may have developed by increasing the number of newly participating genes to this system therefore enhancing its function.

Another possible explanation is that different sets of genes could be involved in memory formation in invertebrates compared to mice. However, it is worth mentioning that memory formation in the mouse hippocampus and *Aplysia*, for example, has common cellular and molecular mechanisms that were highly conserved during evolution^23^. Although the storage of short-term memory in mice and Aplysia requires different signaling, long-term storage of both species utilizes a core signaling pathway. Further components are likely recruited at least in a mouse^3,24^.

In this study, we collected genes related to memory systems without specifying the system considering that memory systems vary among different species. Although, the molecular mechanisms and genes involved in explicit and implicit memory have been studied in some vertebrates and invertebrates; however, due to a lack of information regarding the evolution of memory systems using genome-wide approaches, it is still unknown if there are specific genes associated with the emergence of certain memory systems^3^. More experimental works should be done to determine whether the emergence of specific memory-related genes correlates with the formation of specific memory systems.

Similarly, Fig. 1 shows the number of LMNP orthologous genes increases in vertebrates compared to other species. Remarkably, mice conserve 386 LMNP genes; however, the number of these orthologous genes is slightly reduced in humans. One explanation is that only some of these genes are conserved, and are specific for mice, such as Mex3a, Synj2, Nr1d2, Thbs1, Spred3, Ankrd33b, Chd2, Nup37, Mettl21a, Gdpd5, cml1, Rims1, Efr3a, Armc2, Nfil3, Igsf9, Peg10, Lppr5, Phf13, Lrp5, Ldlr, Stard10, Chrac1, and Stxbp5l. One further explanation could be that humans possess different and undiscovered network genes that may contribute to the LMNP system. The number of orthologous genes decreases slightly in chicken and fish by about 314 and 323 genes, respectively, and the number progressively decreases among invertebrates, choanoflagellates, and yeast, relative to vertebrates (Fig. 1). Thus, the number of orthologous genes of LMNP has consequently increased as a result of evolution.

### Gain and loss comparison of memory and LMNP genes

**Figure 2:**
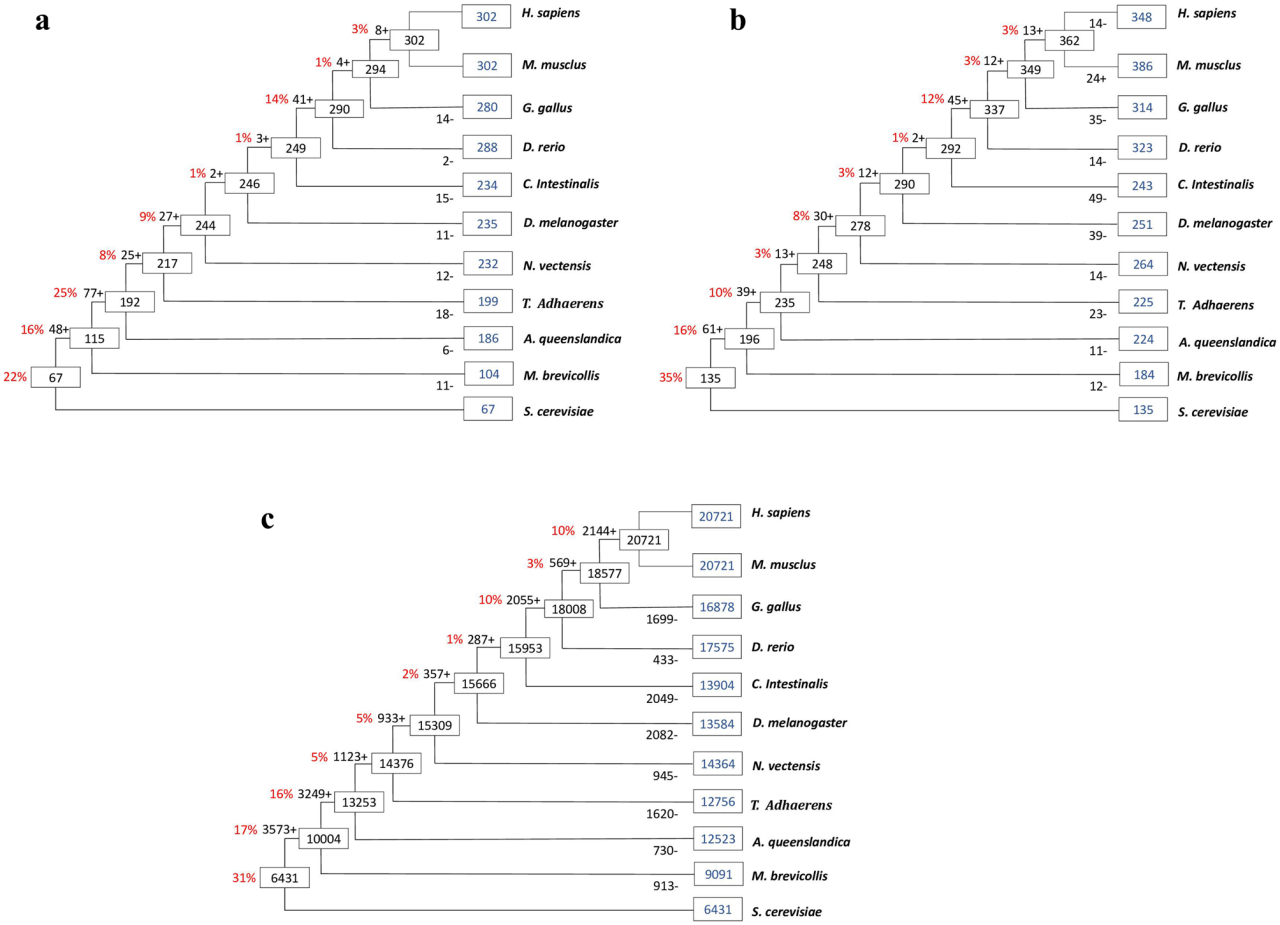
The emergence of the memory (**a**), LMNP (**b**), and reference (**c**) genes during evolution. This schematic tree exhibits gene gain and gene loss events. The blue color represents the number of orthologous genes. Boxes on nodes and numbers on branches show the number of genes present in an ancestor species and gene gains/losses, respectively. Positive marks (+) indicate gene gain, and negative marks (−) display gene loss. The percentage in red color reveals the gene gained during the evolution.

Our analysis shows that 22% of memory-related genes are conserved in yeast, indicating that these genes emerged before the memory function evolved. We identified 67 of 302 orthologous genes in yeast to be related to memory formation, suggesting that yeast is the origin of those genes (Fig. 2a). In contrast, we found that 35% (135 genes) of LMNP orthologous genes are conserved in yeast, which uses lactate as an energy source even before the emergence of neurons, as shown in Fig. 2b. Remarkably, yeast has neither neurons nor a memory system; thus, the original function of these proteins must have altered through evolution since they first emerged.

For example, Gys1 gene is conserved in *S. cerevisiae*, and has function in the synthesis of glycogen^25^. Lsm3 is another protein that is involved in the degradation and splicing of mRNA, the replication of telomere, and the formation of histone^26,27^. Net1 is an essential protein for nucleolar structure in *S. cerevisiae*^28,29^. Margineanu et al.^11^ experimentally examined the involvement of these genes in neural plasticity that is mediated by lactate. Further experimental works are necessary to investigate the exact role of other proteins in yeast and in species that do not possess memory.

Further, we noticed that some genes have gained during the evolution of memory and LMNP systems. For example, the total number of genes conserved in the ancestral species that lack both systems such as *choanoflagellate, A. queenslandica, and T. adhaerens* is 150 (49%) and 113 (29%) genes, respectively (Fig. 2a,b). However, the total number of genes gained in the ancestral species of vertebrates, *C. intestinalis, D. melanogaster, and N. vectensis* is 85 (29%) memory genes and 114 (30%) LMNP genes (Fig. 2a,b). These novel genes must have an essential role in the development of both systems, suggested that improvement of memory in humans may have evolutionarily achieved due to the formation of very sophisticated neural networks in the brain based on those genes.

The number of memory and LMNP-related genes might be affected by the Whole genome duplication (WGD) event. In fact, WGD plays an important role in evolution, particularly significant for the evolution of complex vertebrates^30^, leading to an increase in biological complexity^31^ and facilitating the emergence of evolutionary novelties, a complex set of changes at the genetic and phenotypic level^31–33^. Although WGD increases the genome complexity and supplies raw genetic materials^32^, an extensive gene loss was reported after the WGD event^34,35^.

To clarify the evolutionary processes of both memory and LMNP genes, we used gene gain and loss analysis to detect any tendency in the genes during evolution. We compared the tendency of memory-related genes to the reference, i.e., the gain–loss pattern of the orthologous genes of all the proteins in mice (Fig. 2a,c). We calculated the correlation coefficient of the gene gain patten of the orthologous genes of the memory-related genes and the reference. Consequently, we detected a strong positive correlation (r = 0.814) between memory and the reference.

Similarly, the examination of the tendency of gained LMNP genes helps to understand how these genes evolved. Therefore, we calculated the correlation coefficient of the gene gain pattern of the orthologous genes of LMNP genes and compared it to the gene gain pattern of the reference (Fig. 2b,c). We found a strong positive correlation between the LMNP genes and the reference genes (r = 0.937). Our analysis detected a similar tendency for the gene gain found during the development of the memory system and the LMNP system compared to the reference.

### Evolutionary change of functional categorizations of the orthologs of memory and LMNP proteins

**Figure 3:**
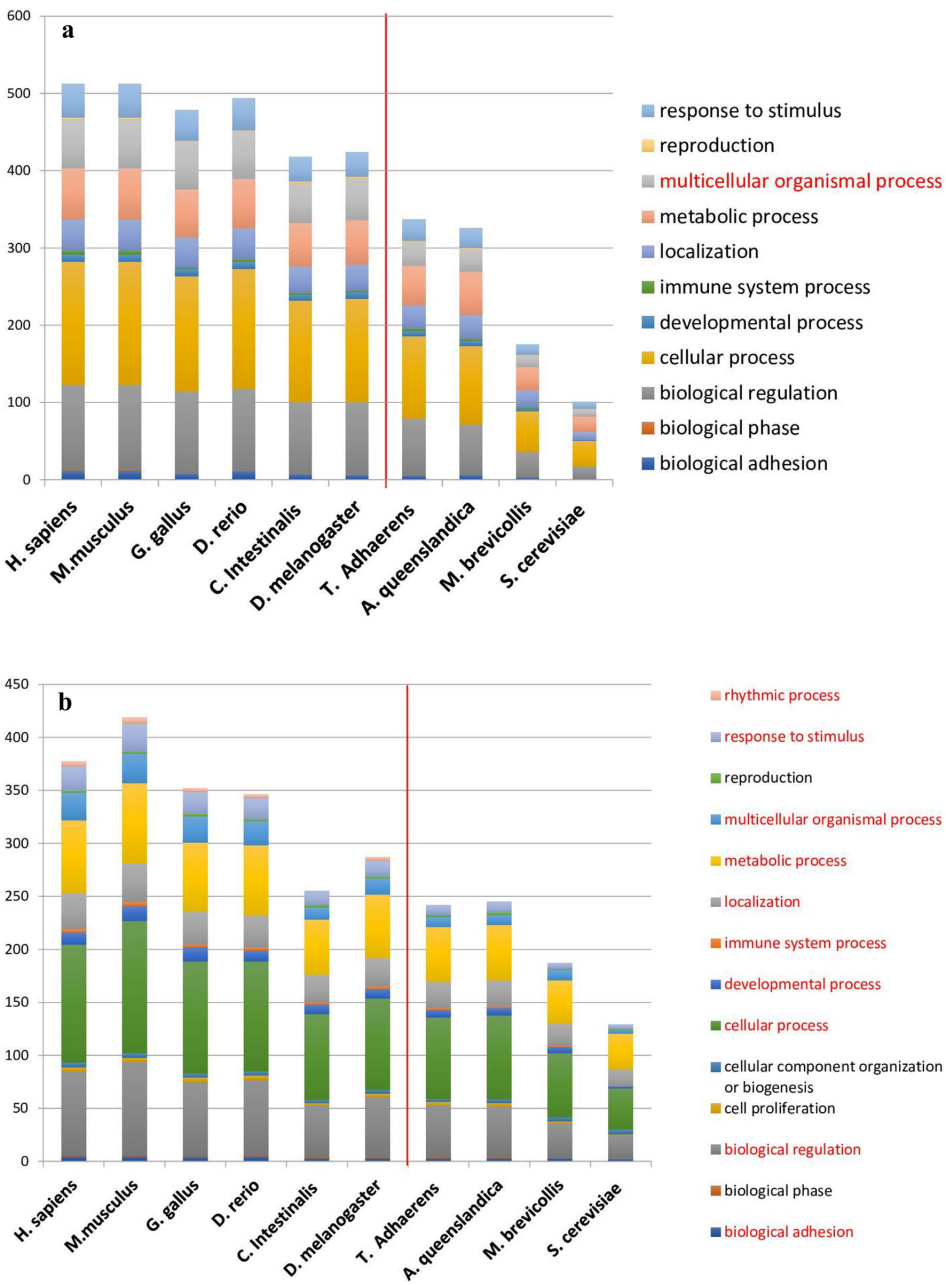
Functional classifications of the orthologs of memory and LMNP proteins. (**a**) Functional groups comparison of memory-related proteins between memory-posses species and memory-lacking species. (**b**) Functional groups comparison of LMNP proteins between LTM- possess species and LTM-lacking species. The red color shows significant categories (*p* < 0.05), and the red line is drawn to divide the two groups visually.

We inferred functional features of memory-related proteins across species using Gene Ontology^36^ and the PANTHER classification system^37^ to examine whether evolutionary changes have occurred in the functional categories during evolution. We performed a statistical analysis [Student’s t-test with multiple test correction (*p* < 0.05)] to examine the differences in the functional categories between two groups: memory-possessing species and memory-lacking species. We found one significant category, which we define as “multicellular organismal processes” (Fig. 3a). To narrow down the functional categories in “multicellular organismal processes”, we analyzed two subcategories, “multicellular organismal development” and “system process”, to explore the significant differences between these two groups. We repeated the analysis on the subcategories up to the fourth layer.

As a result, we obtained “Neurogenesis” as a category, which has a significant difference in the number of orthologous genes between memory-possessing and memory-lacking organisms. Fifteen proteins are related to the “Neurogenesis” category, as shown in Supplementary Table S3, as well as the species distribution of its orthologs. Nine out of 15 proteins are assigned to the “signaling molecule,” “nucleic acid binding,” “transcription factor” and “cytoskeletal protein” at the Molecular Function of Gene Ontology (Supplementary Fig. S1), indicating that the increase of “Neurogenesis” proteins in memory-possessing species may increase the neural networks required for the evolutionary development of the memory system.

A growing body of evidence suggests that lactate participates in long-term potentiation (LTP) and memory formation. LTP is described as the increase in the strength of synapses following the activation of chemical synapses^38^. O'Dowd et al*.*^39^ exhibited that the metabolism of glycogen from astrocytes provided energy for LTM in neonatal chicks, and the administration of glycogen inhibitor 1, 4-dideoxy-1, 4-imino-D-arabinitol (DAB), or glycolysis inhibitor 2-deoxyglucose (2DG) impaired memory performance in chickens^40,41^. Suzuki et al*.*^6^ revealed that glycogen-derived lactate and monocarboxylate transporters (MCTs), i.e., lactate transporters MCT1, MCT2, and MCT4, mainly control LTM formation and LTP, and the blocking glycogenolysis or these transporters causes amnesia and weak synapses, leading to LTP impairment that can be rescued by lactate. Further, Netzahualcoyotzi and Pellerin have shown that decreased expression levels of MCT2 in mature neurons obstruct neurogenesis related to memory consolidation in the hippocampus, suggesting that neuronal MCT2 is required for LTM formation^42^.

To understand how LMNP proteins are involved in the LTM system, we conducted the t-test between LTM-possessing species and LTM-lacking species. LTM is a part of the memory system, and thus the species possessing LTM are the same as memory-possessing species. We performed statistical analysis for the categories and the significant subcategories between these two groups. We identified “biological adhesion," “biological regulation,” “cellular process,” “developmental process,” “immune system process,” “localization,” “metabolic process,” “multicellular organismal process,” “response to stimulus,” and “rhythmic process” as the categories that showed a significant difference between two groups (Fig. 3b). We repeated the analysis on the significant subcategories separately until the fourth level of layers, and we obtained "trans-synaptic signaling," "MAPK cascade," "apoptotic signaling pathway," "neuron development," "generation of neurons," "cellular protein modification process," "translation," "regulation of signal transduction," "regulation of cellular biosynthetic process," " regulation of phosphorus metabolic process," "cell morphogenesis involved in neuron differentiation," "neuron projection development," "embryo development ending in birth or egg hatching," "regionalization," "nervous system development," "neurogenesis," "glycoprotein biosynthetic process," "chordate embryonic development," "anterior/posterior pattern specification," "cellular response to growth factor stimulus," "response to fibroblast growth factor," and "cellular response to peptide” as categories, all of which have significant differences in the number of orthologous proteins between LTM-possessing and non-LTM-possessing organisms.

A total of 86 proteins belong to these significant categories. Species distribution of those orthologous proteins is summarized in Supplementary Table S4. The majority of these proteins (more than ten) are classified as a "signaling molecule" and "transcription factor" (Supplementary Fig. S2). Therefore, the increase in the number of LMNP proteins in LTM-possessing organisms could be required to create complex pathways that enhanced the LMNP function during the evolution.

## Conclusion

Our evolutionary study showed that the memory- and LMNP-related systems seem to have similar evolutionary processes, formed gradually by increasing the number of participating genes, pointing out that these systems never took place at once during evolution. In both systems, a subset of genes had orthologs in yeast, which may be the ancestor of those genes. We found a leaping increase in memory and LMNP genes that took place just before the emergence of vertebrates during evolution. In a similar manner, increasing the number of proteins and their necessary functions among species that possess memory or LTM could be the main reason for the development of both systems. Further, a positive correlation between the gene gain of the memory and LMNP genes was detected once comparing them to the reference, suggesting a similar tendency in the evolutionary gained genes in both systems. Besides, the memory and LMNP systems commonly shared 13 genes; hence, we suggested that the function of LMNP as a signaling system could be evolutionarily linked with the memory system by an unknown system that connects them. Further experimental work should be conducted to examine the function of lactate as a signaling molecule and its association with the memory system among invertebrates to investigate its emergence during evolution. This will help to expand our knowledge to understand more about the involvement of the LMNP system in the evolutionary formation of memory.

## Material and methods

### Identification of memory and LMNP-related genes

We used the PubMed database (https://www.ncbi.nlm.nih.gov/pubmed/) to collect memory-related genes, searching for the keywords Learning AND memory AND (gene OR protein) AND (Mus musculus). We sorted the search results by most recent and customized the selection to include all reviews conducted in the last 19 years (from 1/1/2000 to 19/03/2019). The number of 436 reviews were manually checked and filtered following the criteria that each must contain a list of more than ten genes. After selecting these genes, some were not subtype-specific genes. Those genes have several subtypes; it is not clear which subtype involved in memory formation. To obtain an accurate gene list, a gene that is written in general without determining its subtype was removed from the list. For example, dopamine receptor (Drd) is not specified to subtypes such as Drd1, Drd2, Drd3, Drd4, or Drd5; thus, in this case, we removed it from the list, and so on for all other genes in the list.

LMNP genes were identified by a genome-wide RNA-sequencing study as described in Margineanu et al.^11^. RNA sequencing was performed on RNA isolated from cultured cortical neurons 1 h/6 h after the addition of l-lactate to cell culture media. The expression level of each protein-coding gene was calculated via fragments per kilobase of exon per million mapped fragments (fpkm)^11^. In this study, we collected LMNP genes that were differently expressed (*p* < 0.05) after 1 h and 6 h of lactate treatment^11^and then combined these gene sets (1 h and 6 h) into one list following their definition.

We converted all alias genes into official gene symbols using a python script to compile an accurate gene list and to avoid duplication. We obtained mouse genomic nucleotide accession versions and ID numbers for the all genes from NCBI-nucleotide database and NCBI-gene database, respectively. We extracted amino acid sequences of *M. musculus* for memory and LMNP-related genes from the nucleotide database. For the control, we downloaded the *Mus musculus* proteome from Refseq. We removed all isoforms by selecting the smallest accession number for a protein and we used an alias name for some LMNP proteins (cml1, Lppr5, Diap2, Mfsd4, Pvrl2, and Rp2h) as the sequences were not available using the symbol names.

### Orthologous group identification for memory and LMNP proteins

To detect orthologues, we used the eggNOG (evolutionary genealogy of groups: Non-supervised Orthologous Groups) database version 4.5.1^43^ and the Eggnog mapper tool version 1^44^. We downloaded all 335 memory and 386 LMNP amino acid sequences. The mapping mode was adjusted by default setting (Hmmer). We selected the eukaryotes taxonomic scope to search for mouse memory and LMNP related proteins. We selected all orthologs from the orthologous option, and selected the non-electronic term for the gene ontology evidence.

For the control, the mapping mode adjusted by default setting (Diamond) as hmmer mode did not accept a large number of sequences. We selected and analyzed all proteins that contain one euNOG (eukaryotes) orthologous cluster and excluded those contain more than one orthologous cluster. We wrote a Perl script to detect orthologous proteins among species. Some files were essential for running the script; the first file (euNOG.members.txt.gz) contained eukaryotes non-supervised orthologous groups (euNOG) that were downloaded from eggnog version 3 (http://eggnog.embl.de/version_3.0/downloads.html), and the second file contained the species names and IDs (http://eggnogdb.embl.de/download/latest/eggnog4.core_ species_list.txt).

### Gene gain and loss

We identified the gene gain and the gene loss by count^45^. We used the default parameters for the analysis and analyzed the family history based on the Dollo parsimony.

### Functional classification analysis

We conducted functional classification using the PANTHER functional classification system^37^. We tested the functional classification of the orthologous proteins in all examined species using mouse proteins as reference.

### Statistical analysis

We studied the evolutionary changes of the functional categories of the orthologues of memory and LMNP proteins between species-possess memory (or LTM) such as (*Homo sapiens, Mus musclus, Gallus gallus and Danio rerio, Ciona intestinalis,* and *Drosophila melanogaster*) vs. species-lacking memory (or LTM), including (*Trichoplax adhaerens, Amphimedon queenslandica, Monosiga brevicollis*, and *Saccharomyces cerevisiae)*. We excluded Nematostella from the analysis as it is unknown whether or not it possesses memory. We conducted a Welch’s t-test, applying two-tailed with unequal variance. We applied multiple testing corrections using the Benjamini–Hochberg procedure, and *p* values < 0.05 were considered significant in all analyses.

We calculated the correlation coefficient of the gene gain pattens of the orthologous gene (i.e., a set of absolute numbers of gene gain at each node) of the memory-related genes vs the reference and LMNP-related genes vs the reference using Microsoft Office Excel software.

## Supplementary Information

Supplementary Information.

## Data Availability

The datasets that support the findings of this study is available upon request from the first author [AB].
