# Supplementary material for: Lactate-mediated neural plasticity genes emerged during the evolution of memory systems
Source: Sci Rep. 2022 Nov 10;12:19238. doi: 10.1038/s41598-022-23784-8 (PMC9649800; doi:10.1038/s41598-022-23784-8)

Supplementary Information

Title:

Lactate-Mediated Neural Plasticity genes emerged during the evolution of memory systems

Authors

Amal Bajaffer[1,2], Katsuhiko Mineta[1,3,4], Pierre Magistretti[2], Takashi Gojobori[1,2]

Affiliations

[1] Computational Bioscience Research Center (CBRC), King Abdullah University of Science and Technology (KAUST), Thuwal 23955-6900, Saudi Arabia

[2] Biological and Environmental Science and Engineering division (BESE), King Abdullah University of Science and Technology (KAUST), Thuwal 23955-6900, Saudi Arabia

[3] Computer, Electrical and Mathematical Sciences and Engineering division (CEMSE), King Abdullah University of Science and Technology (KAUST), Thuwal 23955-6900, Saudi Arabia

[4] Research Organization for Nano & Life Innovation, Waseda University, Tokyo 162-0041, Japan

Supplementary Table S1: Literature reviews that used in this study.

#	Title of Review	Date of publication
1	Estrogenic regulation of memory consolidation: A look beyond the hippocampus, ovaries, and females	2018
2	Epigenetic regulation of estrogen-dependent memory	2014
3	Genes and signaling pathways involved in memory enhancement in mutant mice	2014
4	Epigenetics, estradiol, and hippocampal memory consolidation	2013
5	Neuropeptides in learning and memory	2013
6	The ubiquitin–proteasome system as a critical regulator of synaptic plasticity and long-term memory formation	2013
7	Hippocampus development and function: role of epigenetic factors and implications for cognitive disease	2010
8	The molecular and cellular biology of enhanced cognition	2009
9	Common molecular mechanisms in explicit and implicit memory	2006
10	Gene targeting of presynaptic proteins in synaptic plasticity and memory: Across the great divide	2006
11	Early genomics of learning and memory: a review	2005
12	Genetic Dissection of Learning and Memory in Mice	2004
13	Learning, Memory, and Transcription Factors	2003
14	The Role of PKA, CaMKII, and PKC in Avoidance Conditioning: Permissive or Instructive?	2002

Supplementary Table S2: The orthologs of common proteins between memory and LMNP systems. The presence and absence of orthologs indicate 1 and 0, respectively.

	H. sapiens	***M.musculus***	***G. gallus***	***D. rerio***	***C. Intestinalis***	***D. melanogaster***	***N. vectensis***	***T. Adhaerens***	***A. queenslandica***	***M. brevicollis***	***S. cerevisiae***
Arc	1	1	1	0	0	0	0	0	0	0	0
Junb	1	1	1	1	1	1	1	1	1	0	1
Bdnf	1	1	1	1	0	0	0	0	0	0	0
Kcna6	1	1	1	1	1	1	1	1	0	1	0
Egr1	1	1	1	1	1	1	1	1	1	1	1
Nr4a1	1	1	1	1	1	1	1	0	0	0	0
Atf4	1	1	1	1	1	1	0	0	1	0	0
Fos	1	1	1	1	1	1	1	1	1	1	1
Itga3	1	1	0	1	0	0	0	0	0	0	0
Bcl2l11	1	1	0	0	0	0	0	0	0	0	0
Hcn1	1	1	1	1	1	1	1	1	0	0	0
Adcyap1	1	1	1	1	0	0	0	0	0	0	0
Cckbr	1	1	1	1	1	1	1	1	1	0	0

Supplementary Table S3. The orthologous proteins of “Neurogenesis”. The presence and absence of orthologs indicate 1 and 0, respectively.

**Protein Name**	***H. sapiens***	***M.musculus***	***G. gallus***	***D. rerio***	***C. Intestinalis***	***D. melanogaster***	***T. Adhaerens***	***A. queenslandica***	***M. brevicollis***	***S. cerevisiae***
Pak1	1	1	1	1	1	1	1	1	1	1
Bdnf	1	1	1	1	0	0	0	0	0	0
Gap43	1	1	1	1	0	0	0	0	0	0
Adcyap1	1	1	1	1	0	0	0	0	0	0
Fxr2	1	1	1	1	1	1	0	1	1	0
L1cam	1	1	1	1	1	1	1	1	1	0
En2	1	1	1	1	1	1	0	0	0	0
Bsg	1	1	1	1	1	0	0	0	0	0
Ntf5	1	1	0	0	0	0	0	0	0	0
Map2	1	1	1	1	0	1	0	0	0	0
Mapt	1	1	1	1	0	1	0	0	0	0
Map1b	1	1	1	1	0	1	0	1	1	0
Ngf	1	1	1	1	0	0	0	0	0	0
Rb1	1	1	1	1	1	1	1	1	0	0
Fmr1	1	1	1	1	1	1	0	1	1	0

Supplementary Table S4: The orthologous distribution of 86 of LMNP proteins. The presence and absence of orthologs indicate 1 and 0, respectively.

Protein name	*H. sapiens*	*M.musculus*	*G. gallus*	*D. rerio*	*C. Intestinalis*	*D. melanogaster*	*N. vectensis*	*T. Adhaerens*	*A. queenslandica*	*M. brevicollis*	*S. cerevisiae*
Vav2	1	1	1	1	1	1	1	1	1	1	0
Eif4ebp1	1	1	1	1	1	1	1	1	1	0	0
Emd	1	1	0	1	0	0	0	0	0	0	0
Hes5	1	1	1	1	0	0	1	0	0	0	0
Amy1	1	1	1	1	1	1	0	0	0	1	0
Nr1d2	0	1	0	0	0	0	0	0	0	0	0
Plp1	1	1	1	1	1	1	0	0	0	0	0
Penk	1	1	1	1	0	0	0	0	0	0	0
Med26	1	1	1	1	1	1	1	1	1	1	1
Smad3	1	1	1	1	1	1	1	1	1	0	0
Chpf2	1	1	1	1	1	1	1	0	0	0	0
Prkca	0	1	0	1	1	1	1	1	0	1	0
Pkn1	1	1	1	1	1	1	1	1	1	1	1
Spry2	0	1	1	1	1	1	0	0	0	0	0
Trib1	1	1	1	1	1	1	1	1	1	1	1
Pim3	1	1	1	1	1	1	1	1	1	1	1
Scn1a	1	1	1	1	0	1	1	0	0	1	0
Gadd45g	1	1	0	1	0	0	1	0	0	0	0
Sertad3	1	1	0	0	0	0	0	0	0	0	0
Gadd45b	1	1	1	1	0	0	0	0	0	0	0
Ghr	1	1	1	1	0	0	0	0	0	0	0
Rcan2	1	1	1	1	1	1	1	1	1	1	1
Cryl1	1	1	1	1	1	1	1	0	1	1	0
Arid5a	0	1	0	1	0	0	0	0	0	0	0
Cars	1	1	1	1	1	1	1	1	1	1	1
Arc	1	1	1	0	0	0	0	0	0	0	0
Peli3	1	1	1	1	1	1	1	1	1	0	0
Pthlh	1	1	1	1	0	0	0	0	0	0	0
Taf9b	1	1	1	1	1	1	1	1	1	0	1
Junb	1	1	1	1	1	1	1	1	1	0	1
Fgf11	1	1	1	1	1	1	1	0	0	0	0
Sgtb	1	1	1	1	0	1	0	0	1	1	1
Id2	1	1	1	1	0	1	0	0	0	0	0
Ring1	1	1	1	1	0	1	1	1	1	1	0
Bdnf	1	1	1	1	0	0	0	0	0	0	0
Spry4	1	1	1	1	0	0	1	0	0	0	0

Ttbk1	1	1	1	1	1	1	1	1	1	1	1
Lpin1	1	1	1	1	1	1	1	1	1	1	1
cml1	0	1	0	0	0	0	0	0	0	0	0
Hmgb2	1	1	1	1	1	1	1	1	1	1	1
Glyctk	1	1	1	1	1	1	1	1	1	1	0
Rims1	0	1	0	0	0	0	0	0	0	0	0
Rims4	1	1	0	0	0	0	0	0	0	0	0
Slitrk4	1	1	1	1	1	0	0	0	0	0	0
Map3k11	1	1	1	1	1	1	1	1	1	1	0
Hrk	1	1	0	0	0	0	0	0	0	0	0
Npas4	1	1	0	1	1	1	0	0	0	0	0
Nr4a3	1	1	1	1	1	1	1	0	0	0	0
Nr4a1	1	1	1	1	1	1	1	0	0	0	0
Nr4a2	1	1	1	1	1	1	1	0	0	0	0
Epha10	1	1	1	1	1	1	1	1	1	1	0
Mrpl55	1	1	0	1	1	1	1	0	0	0	0
Nfil3	0	1	0	0	0	0	0	0	0	0	0
P3h3	1	1	1	1	0	0	1	0	1	0	0
Ssu72	1	1	1	1	1	1	1	1	1	1	1
Pars2	1	1	1	1	1	1	0	1	1	1	1
Pkib	1	1	0	1	0	0	0	0	0	0	0
Rgs4	1	1	1	1	1	1	1	1	1	1	1
Tbr1	1	1	1	1	1	1	1	1	1	0	0
Per3	1	1	1	1	0	1	0	0	0	0	0
Per2	1	1	1	1	0	1	0	0	0	0	0
Diras2	1	1	1	1	1	1	1	1	1	1	1
Cacna1i	1	1	0	1	0	0	1	0	0	0	0
Sltm	0	1	1	0	0	0	1	0	0	0	0
Adgrg1	1	1	1	1	1	1	1	1	1	1	0
Gfra2	1	1	1	1	0	0	0	0	0	0	0
Dcun1d3	1	1	1	1	1	1	0	1	1	1	1
Extl1	1	1	1	1	1	1	1	1	1	1	0
Dusp6	1	1	1	1	1	1	1	1	1	1	1
Dusp4	1	1	1	1	1	1	1	1	1	1	1
Dusp10	1	1	1	1	1	1	1	1	1	1	1
Vegfa	1	1	1	1	0	0	0	0	0	0	0
Jup	1	1	1	1	1	1	1	1	1	1	1
Madd	1	1	1	1	1	1	1	1	1	0	0
Adcy8	1	1	1	1	1	1	1	1	1	0	0
Sema3e	1	1	1	1	1	1	1	1	1	1	0
Sema4b	1	1	1	1	1	1	1	1	1	1	0
Sema6c	1	1	1	1	1	1	1	1	1	1	0
Igfbp3	1	1	1	1	0	0	0	0	0	0	0
Adcyap1	1	1	1	1	0	0	0	0	0	0	0
Per1	1	1	0	0	0	0	0	0	0	0	0
Ispd	1	1	1	1	1	0	1	1	0	1	0

Numb	1	1	1	1	1	1	1	1	0	0	0
Htr7	1	1	1	1	1	1	1	1	1	0	0
Gpr26	1	1	1	1	1	1	1	1	1	0	0
Htr5a	1	1	1	1	1	1	1	1	1	0	0

Supplementary Fig. S1: Molecular function of the "Neurogenesis" proteins.

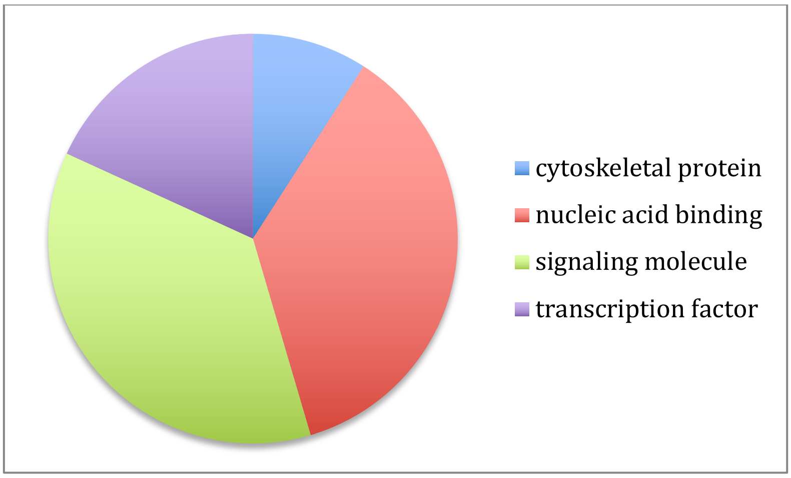

Supplementary Fig. S2: Molecular function of the LMNP proteins resulted from the 4th level significant categories.

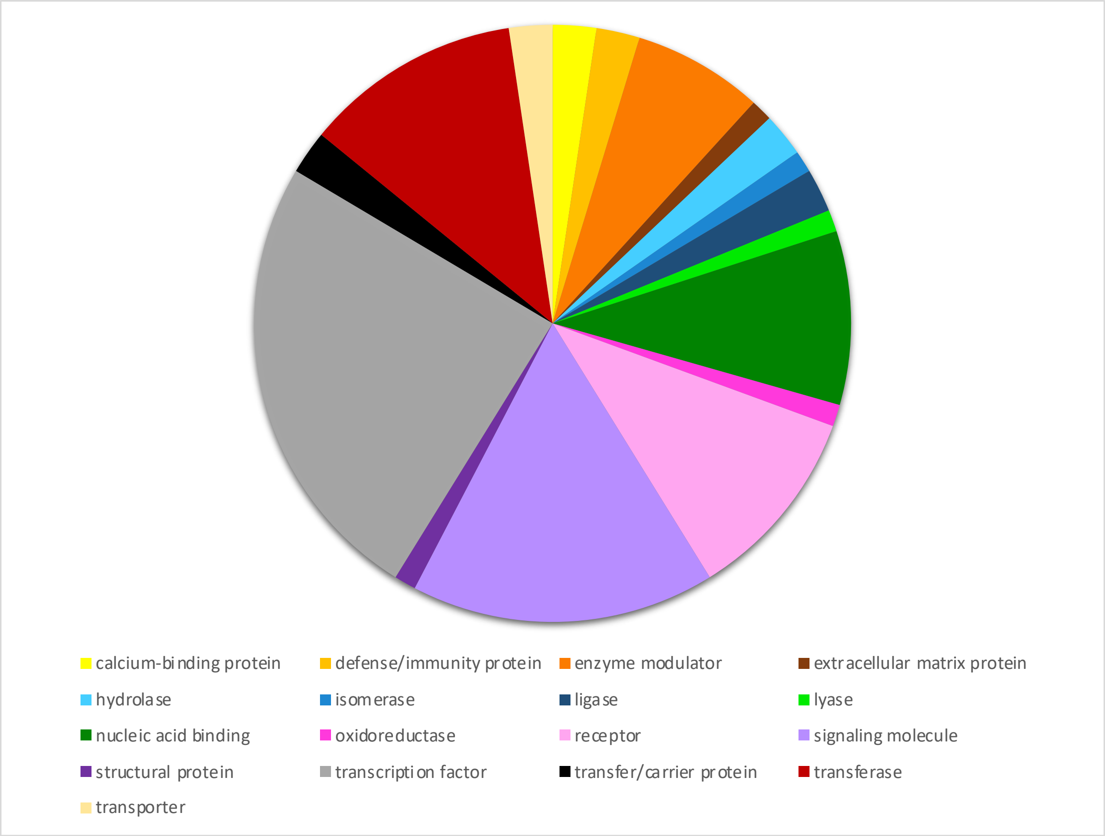

Supplement: Supplementary file 1 — Supplementary Information. [file 41598_2022_23784_MOESM1_ESM.pdf]
